# Executive Clemency—Pardon of Dr. Beale

**Published:** 1856-01

**Authors:** 


### EXECUTIVE CLEMENCY—PARDON OF DR. BEALE.

Gov. Pollock has extended his clemency to Dr. Beale, and remitted the remainder of his sentence of imprisonment, which was four years and six months, beginning on the 28th of November, 1854. He has served, therefore, about one year of his term. The pardon states the reasons which induced the Governor to extend this favor.

He had received communications from about one hundred and forty dentist and twenty-three physicians, of this city and the country, stating their belief that testimony as to matters transpiring under the influence of ether is unsafe and unreliable; from a number of other physicians named, that they believed him innocent; from a number of the bar, and citizens of various states, including the names of Governors, Attorneys-General, etc., that they believed he was convicted on insufficient testimony; from a number of clergymen, that they believed him innocent; from the Mayor of Philadelphia, and fifty members of the Philadelphia City Council; from members of the Legislature, Judges of the Supreme Court; Editors of Philadelphia newspapers, and five thousand other citizens of Pennsylvania and New-York, with five of the jury on the trial, all asking his pardon. After enumerating all these facts, the Governor says:

"And whereas, the Board of Inspectors of the said Philadelphia County Prison, (as appears by their communication on file in the office of the Secretary of the Commonwealth), have unanimously recommended the pardon of the said Dr. Stephen T. Beale, because, in their opinon, the end contemplated by the law in the moral reform of the prisoner has been attained—because full and ample satisfaction has been rendered to public sentiment by the imprisonment he has already undergone—because his health is undoubtedly breaking down under the sufferings of body and mind which he has already endured, and because the destitute condition of his aged parents and bereaved and sorrowing wife and children imperatively demand the presence and support of their son, husband and father.

"And whereas, after a full and careful examination of the facts and evidence in the case, aided by the scientific discussion to which it has given rise, (without any intention to reflect upon the prosecutrix, who no doubt testified to what she believed did occur—nor to impugn the integrity of the learned Judge who tried the case, nor the honesty of the jury who convicted the prisoner), *I am now satisfied* that the defendant, Dr. Stephen T Beale, is *not guilty* of the crime whereof he stands charged, and was convicted upon evidence unreliable in its character and insufficicnt in amount.

"I do, therefore, in consideration of the premises, pardon the said Dr. Stephen T. Beale of the crime whereof he is convicted as aforesaid, and he is hereby fully pardoned accordingly."

We have no disposition to throw odium on the trial by jury, God forbid—but that such a low-lived, vulgar, *drunken* jury—singing the "Landlady of France," etc., should be allowed to "scorch him," or to act on a charge against any man but one of their own peculiar class, we cannot understand. How a respectable and exemplary bench should permit such a verdict under the circumstances, is a wonder not only to us, but to thousands of others. One of the oldest and most respected medical practitioners of our city, said to us, that this was the first in the annals of his memory, that made him doubt the stability of our institution, and absolutely tremble for his own personal freedom. We say, unhesitatingly, that no man who drinks intoxicating liquors while in the jury-box, except for disease, is a fit person to decide quistions involving the characters and lives of his fellow men.—*Phil. Med. and Surg. Jour.*

